# Epigenetic Changes in Alzheimer’s Disease: DNA Methylation and Histone Modification

**DOI:** 10.3390/cells13080719

**Published:** 2024-04-21

**Authors:** Laura Maria De Plano, Alessandra Saitta, Salvatore Oddo, Antonella Caccamo

**Affiliations:** Department of Chemical, Biological, Pharmaceutical and Environmental Sciences, University of Messina, Viale F. Stagno d’Alcontres 31, 98166 Messina, Italy; alessandra.saitta@unime.it (A.S.); salvatore.oddo@unime.it (S.O.)

**Keywords:** APP, tau, DNA methylation, Aβ, histone modification

## Abstract

Alzheimer’s disease (AD) is a devastating neurodegenerative disorder characterized by progressive cognitive decline and memory loss, imposing a significant burden on affected individuals and their families. Despite the recent promising progress in therapeutic approaches, more needs to be done to understand the intricate molecular mechanisms underlying the development and progression of AD. Growing evidence points to epigenetic changes as playing a pivotal role in the pathogenesis of the disease. The dynamic interplay between genetic and environmental factors influences the epigenetic landscape in AD, altering gene expression patterns associated with key pathological events associated with disease pathogenesis. To this end, epigenetic alterations not only impact the expression of genes implicated in AD pathogenesis but also contribute to the dysregulation of crucial cellular processes, including synaptic plasticity, neuroinflammation, and oxidative stress. Understanding the complex epigenetic mechanisms in AD provides new avenues for therapeutic interventions. This review comprehensively examines the role of DNA methylation and histone modifications in the context of AD. It aims to contribute to a deeper understanding of AD pathogenesis and facilitate the development of targeted therapeutic strategies.

## 1. Introduction

Alzheimer’s disease (AD) is the most common neurodegenerative disorder that afflicts the elderly. The probability of developing AD doubles every 5 years after the age of 65 and there is a 50% chance of being diagnosed with AD after the age of 85 [1,2]. It is estimated that almost 44 million people currently suffer from dementia. This number is projected to triple by 2050. AD is the major cause of dementia, accounting for 50–75% of these cases worldwide [3]. More than 90% of AD forms are sporadic and occur in people older than 65 years, while the remaining cases are familial or early-onset AD and can manifest in people between the ages of 30 and 65 [1,2]. While the causes of sporadic AD are unknown, growing evidence indicates that it is triggered by a complex interplay between genetic and environmental factors. The major risk factors are age and the presence of the E4 allele of the apolipoprotein E (*APOE*) gene [1,2]. The familial cases (FAD) are due to highly penetrant mutations in three genes, the amyloid precursor protein (*APP*), presenilin 1 (*PSEN1*), and presenilin 2 (*PSEN2*) [1,2]. It is well established that mutations in these genes lead to the accumulation and aggregation of the β-amyloid (Aβ) protein [1,2].

Clinically, AD is characterized by a spectrum of clinical manifestations encompassing cognitive, functional, and behavioral dimensions [2]. Individuals with mild dementia manifest significant changes in multiple cognitive and behavioral functions, including memory loss and language difficulties [4]. With the progression of the disease, they start to manifest impaired judgment, disorientation, and confusion. Minor clinical manifestations include severe behavioral changes, such as aggression, agitation, and delusions. Later in the disease, impaired mobility, hallucinations, and seizures could manifest until death, which occurs on average 8.5 years after the presentation of the most severe symptoms [1,2]. Generally, death in AD patients occurs for secondary reasons such as pneumonia. The heterogeneous nature of symptom progression emphasizes the need for early detection and intervention to enhance the overall quality of life for individuals with Alzheimer’s and their caregivers.

The major hallmarks associated with AD pathology are plaques and neurofibrillary tangles (NFTs) [1,2]. Plaques are extracellular deposits made primarily of Aβ, a peptide comprised of 40–42 amino acids. Aβ derives from the cleavage of a larger protein, the amyloid precursor protein (APP) located on chromosome 21 [5]. APP is commonly cleaved through two different pathways: the non-amyloidogenic and amyloidogenic pathways. In the non-amyloidogenic pathway, APP is cleaved first by α-secretase that cuts in the middle of the Aβ peptide, yielding a soluble and non-pathogenic precursor, C83, and a larger fragment known as αAPP, which has neuroprotective properties [5]. Subsequently, C83 is cut by β-secretase to generate a small hydrophobic fragment p3 that has a role in synaptic signaling [5]. To generate Aβ, APP is cut first by β-secretase, also known as β-site APP-cleaving enzyme 1. This enzyme cleaves APP at the N-terminal region of Aβ, generating a larger N-terminal fragment, βAPP, and C99, a fragment that contains the entire Aβ peptide. To free Aβ, C99 is subsequently cut by β-secretase, liberating the C-terminal fragment known as APP intracellular domain and Aβ [5]. Depending on the cleavage site of the β-secretase, Aβ could be 40 or 42 amino acids long. Aβ42 is less abundant, but highly insoluble, neurotoxic, and more prone to aggregate [5]. The other pathological feature present in the brain of AD patients is NFTs, filamentous extracellular aggregates made of hyperphosphorylated tau protein. In physiological conditions, tau, a protein encoded by the *MAPT* gene located on chromosome 17, is a soluble protein that promotes microtubule assembly and stabilization [5,6]. In AD, however, tau becomes insoluble and aggregates, forming filamentous structures. In addition, pathological tau is abnormally phosphorylated at specific residues reducing its affinity for microtubules and leading to neurotoxicity and neurodegeneration [7].

Inflammation is another neuropathological hallmark of AD brains. Growing evidence suggests a self-perpetuating cycle between the accumulation of Aβ and microglia activation [8]. To this end, microglia, the resident immune cells in the brain, become activated in response to Aβ accumulation. In turn, activated microglia release pro-inflammatory cytokines, such as interleukin-1β (IL-1β) and tumor necrosis factor-alpha (TNF-α), exacerbating the inflammatory response and contributing to Aβ production [8]. A similar crosstalk has been found for microglia and tau. Proinflammatory cytokines can activate several protein kinases which in turn can phosphorylate tau and promote its aggregation. Concomitantly, the accumulation of NFTs trigger microglia activation and further release of pro-inflammatory cytokines [8]. Overall, there is an intricate relationship between neuroinflammation, and AD neuropathology and several laboratories are actively studying these processes to gain a better understanding of the underlying mechanisms.

Synaptic degeneration and cell death represent another invariable feature of AD brains. Synaptic degeneration might manifest before neuronal loss and could contribute to the cognitive deficits associated with AD patients [9]. Loss in neurons begins in the early stages in the entorhinal cortex, basal nucleus of Meynert, and in the locus coeruleus but later spreads in multiple areas like the hippocampus, amygdala, and cortex [9]. While the mechanisms that cause cell death in AD brains are poorly understood, strong evidence points to the buildup of Aβ as a key factor [10,11,12]. Along these lines, overwhelming evidence has indicated that accumulation of Aβ can trigger tau pathology, which in turn can directly or indirectly lead to synaptic dysfunction, neuronal death, and memory deficits [12,13,14]. Loss of synapses is the best neuropathological correlate with cognitive decline [15].

Currently, there are no effective treatments for AD. The most prescribed pharmacological approach is the use of cholinesterase inhibitors, which include donepezil, galantamine, and rivastigmine. Mechanistically, these interventions aim to compensate for the death of cholinergic neurons. They lead to temporary symptomatic improvements and as such generally they lose their efficacy rather quickly as the disease progresses. However, these approaches target the symptoms more than the root of the disease and as such generally lose their efficacy rather quickly. Memantine, an NMDA-receptor agonist, is usually prescribed for moderate-to-severe AD, but it is characterized by severe side effects. More recently, the U.S. Food and Drug Administration has approved two monoclonal antibodies for AD, aducanumab and lecanemab, which have been shown to reduce Aβ deposits in some patients [16,17,18]. However, serious safety concerns for these therapies have been reported and further studies are needed to address whether the benefits outweigh the risks [19].

In this review, we initially delve into the various approaches employed for evaluating DNA methylation and histone modification. Subsequently, we elaborate on the interconnection between these epigenetic alterations and AD pathogenesis. Finally, we explore potential avenues for therapeutic interventions. We focused primarily on original research articles investigating epigenetic alterations in human AD tissue. We also considered all the studies that combined the research on human samples with animal and cell models.

## 2. Available Techniques to Assess DNA Methylation and Histone Modification

The different techniques used to assess DNA methylation and histone modification have been reviewed elsewhere [20]. Here, we report the basic principles, focusing on their advantages and disadvantages. 

Bisulfite sequencing is a widely used technique in epigenetics research to assess DNA methylation. The process involves the treatment of DNA with sodium bisulfite, which converts unmethylated cytosine residues to uracil while leaving methylated cytosines unchanged [21]. By analyzing the sequence data obtained from bisulfite-treated DNA, one can determine the methylation status of cytosine residues at single-base resolution. This level of resolution represents the major advantage of this technique together with the fact that it can be applied to both targeted regions and whole-genome analysis. However, bisulfite sequencing has its drawbacks, which include the following: (i) by converting unmethylated cytosines to uracil, one can miss small nucleotide polymorphisms; (ii) it does not distinguish between 5-methylcytosine and 5-hydroxymethylcytosine; (iii) it can be time-consuming and costly; (iv) often some regions of the DNA can be over- or underrepresented; (v) DNA degradation can occur [22]. Different variations of bisulfite sequencing are now available, which include whole-genome bisulfite sequencing and reduced representation bisulfite sequencing. The former is very similar to bisulfite sequencing but applies to the entire genome. The latter combines bisulfite treatment with restriction enzyme digestion to selectively sequence regions of the genome enriched for CpG sites [21,23].

Methylation-specific PCR is a sensitive and specific method for detecting DNA methylation at specific loci, making it particularly useful for analyzing DNA methylation patterns in regions of interest, such as gene promoters or CpG islands [24]. It relies on the differential sensitivity of methylated and unmethylated cytosines to treatment with sodium bisulfite, followed by PCR amplification using primers designed to specifically target either methylated or unmethylated DNA sequences. However, it is important to note that MSP is limited to the detection of known methylation sites and may not provide comprehensive information about DNA methylation across the entire genome [24]. 

Methylated DNA immunoprecipitation sequencing (MeDIP-seq) is a technique used to investigate DNA methylation patterns at a genome-wide scale [25]. As its name implies, it combines immunoprecipitation with high-throughput sequencing to identify regions of DNA that are methylated. Compared to other methods such as bisulfite sequencing, MeDIP-seq is less affected by DNA degradation and can be applied to relatively small amounts of input DNA. However, it provides lower resolution information compared to techniques like bisulfite sequencing, as it identifies methylated regions rather than individual methylated cytosines. Another drawback is that it depends on specific antibodies against methylated DNA [25].

Chromatin immunoprecipitation (ChIP) is a versatile technique that has revolutionized our understanding of gene regulation and chromatin dynamics by providing insights into the localization and function of proteins within the chromatin landscape [26]. It is widely used to study the roles of transcription factors, histone modifications, chromatin remodeling complexes, and other chromatin-associated proteins. The first step involves cross-linking histones to DNA to preserve the interactions. The histone/DNA complexes are then immunoprecipitated and the cross-linkages are reversed by acid or increased temperature. Histones and DNA can then be analyzed using multiple downstream methods [26]. This approach allows the study of specific histone modifications at selective genomic loci. However, it requires a large number of cells, cross-linking reactions which can damage the DNA and selective antibodies. As such, the background noise can often be high. 

Mass spectrometry has been used for direct measurement of histone modification. The major advantage of this approach is that it provides quantitative data, but it requires high technical expertise both for the extraction and purification of the histones and the actual spectrometry analysis [27]. 

## 3. DNA Methylation in AD

DNA methylation is an epigenetic mechanism involved in regulating gene expression. DNA is methylated by a family of enzymes known as DNA methyltransferases (DNMTs) [28]. These enzymes start a one-carbon metabolism cycle, where S-adenosylhomocysteine is converted back to S-adenosylmethionine, after which a methyl group is transferred to the DNA. The production of homocysteine, an intermediate byproduct of the methylation reaction, is associated with a higher risk of AD and cerebrovascular disease [29,30]. Generally, DNA methylation occurs at CpG sites in the genome, and it is estimated that close to 90% of the CpG sites are methylated. Indeed, 5-methylcytosine is the most abundant modified base in the mammalian genome. Nonetheless, recent studies have identified that other bases can also be methylated (e.g., N6-methyladenine and 5-formylcytosine [31].

Among the DNA methyl transferases, DNMT1 is the main enzyme responsible for the maintenance of the DNA methylation signatures during cell division and is implicated in regulating gene expression and maintaining genome stability. Dysregulation of DNMT1 activity has been observed in various neurodegenerative diseases. In AD, DNMT1 protein levels are decreased in the hippocampal and temporal brain region but increased in the frontal cortex, temporal cortex, and cerebellum [32,33,34]. Importantly, the precise mechanisms underlying the involvement of DNMT1 in AD are still under investigation, and most likely, the relationship between DNMT1 dysregulation and AD pathogenesis is multifaceted. Future research is needed to elucidate the specific genes and pathways that DNMT1 regulates in AD and to determine whether targeting it could represent a potential therapeutic strategy.

### 3.1. Global DNA Methylation

Several research groups have analyzed global DNA methylation in various brain regions of a sample population with and without AD. A careful analysis of the literature indicates that contradictory reports have been published on whether global DNA methylation is altered in AD brains. Early reports indicate that global DNA methylation in the entorhinal cortex and hippocampus of postmortem AD brains is reduced [35,36]. However, others have reported an overall increase in DNA methylation in AD [32,37,38,39]. Yet, other studies have found no changes between AD and controls [40,41]. The inconsistency in these reports seems to extend beyond the methods used to measure DNA methylation or the specific brain regions in which the measurements were taken. For example, Chouliaras and colleagues and Bradley-Whitman and colleagues published opposite results related to the methylation state of neurons in the hippocampus of AD cases. The former reported a significant decrease in DNA methylation in AD, while the latter reported a significant increase [36,38]. More studies are needed to resolve these inconsistencies, and perhaps the various groups studying DNA methylation in AD should standardize the techniques employed, the quality of the tissue, and its processing. A similar attempt has been made to measure autophagy [42].

The relation between AD neuropathology and changes in DNA methylation is another unresolved issue. It has been reported that 5 hmC levels are significantly higher in the middle frontal gyrus and middle temporal gyrus of AD brains compared to age-matched controls. The authors also reported a positive correlation between high 5 hmC levels and NFTs [39]. Consistent with these data, 17 differentially methylated positions in the blood of AD patients were correlated with higher tau pathology [43]. However, others have reported that global DNA methylation is reduced in tangle-positive neurons [44]. Chouliaras and colleagues measured the hippocampal levels of 5-mC and 5-hmC in AD and age-matched control cases. They reported that in AD, there was a ~20% decrease for both markers. Notably, they found a significant inverse correlation between these two markers and hippocampal Aβ load, while reporting no correlation between 5-mC and 5-hmC levels and NFTs [36]. In a similar study, Coppieters and colleagues measured 5-mC and 5-hmC in human middle frontal and temporal gyri. In contrast with the report by Chouliaras and colleagues, they reported that 5-mC and 5-hmC levels were increased in AD brains compared to age-matched control cases [39]. Furthermore, they reported a positive correlation between 5 mC and 5 hmC levels and Aβ and tau [39]. While the number of patients analyzed in these two studies is relatively low (<30 in both studies), the data are in clear conflict. In a state-of-the-art study, Shireby and colleagues analyzed genome-wide DNA methylation in dorsolateral prefrontal and occipital cortexes of over 600 AD patients [45]. They found 67 differentially methylated positions associated with AD. Of these, 22 showed an increase in methylation and a positive correlation with NFTs. Similarly, 14 hypermethylated sites showed a positive correlation with Aβ load [45]. 

While the apparent differences between these results remain elusive, it is tempting to speculate that post-mortem interval and tissue processing may alter DNA methylation and thus account for some of these inconsistencies [46]. In addition, it has also been suggested that changes in DNA methylation may result from the varying proportions of cell types in the AD brain. With the progression of the disease, neurodegeneration may affect the numbers of neurons and glial cells differently, and these changes may vary from case to case. Therefore, the fluctuations in cell proportions could potentially confound DNA methylation studies [47,48].

The methylation of mitochondrial DNA has also been highly investigated. Work in animal models and a small number of postmortem human brains indicated an increase in methylation of the D-loop region of mitochondrial DNA, isolated from the entorhinal cortex, early in the progression of the disease [49,50]. In contrast, in peripheral blood, methylation levels of the D-loop of mitochondrial DNA appear to be reduced. Indeed, Stoccoro and colleagues, using blood from over 260 people (133 AD and 130 controls), reported a 25% reduction in mtDNA D-loop methylation levels [51]. The same group also reported that in contrast, methylation levels of the mitochondrial DNA’s D-loop were increased in mild cognitive impairment. As for global DNA methylation, further work is needed to better elucidate the relationship between mitochondrial DNA methylation and disease progression.

### 3.2. Gene-Specific DNA Methylation

Another approach to studying the involvement of DNA methylation in AD pathogenesis is to focus on the methylation status of specific genes. This targeted approach has highlighted the role of individual genes in AD pathogenesis. Generally, the methylation of CpG sites at the 5′ promoter region is associated with reduced transcription, whereas methylation of CpG sites in other regions of the gene could be associated with enhanced transcriptional activity [52]. In addition, low methylation levels in enhancers and promoters usually result in an increased expression of the target gene, which leads to an activation of apoptotic and inflammatory pathways in AD [53]. In AD, several studies have reported that the methylation status of *APP*, *PSEN1*, *BACE1*, *MAPT*, and *APOE* genes may be altered [54]. 

#### 3.2.1. APP

While the involvement of APP in the pathogenesis of AD is undisputed, more needs to be done to characterize the methylation state of its gene as apparent contradicting reports have been published. To this end, it has been reported that the *APP* promoter is hypomethylated in neurons and glia of AD patients [55,56]. However, others have reported hypermethylation of the *APP* gene, which was associated with increased expression in both neuronal and non-neuronal cells in the temporal cortex of AD. However, others have reported no differences in the percentage of CpG methylation of *APP* in the frontal cortex and hippocampus of AD patients [57]. It is not clear if these apparent discrepancies are due to technical differences among the groups of the status of the conserved human brain. To this end, Jarmasz and colleagues reported that DNA methylation is stable for about 72 h post-mortem [58]. These findings underscore the significance of normalizing the postmortem interval of tissue when comparing methylation studies from different groups.

#### 3.2.2. PSEN1

Several studies have investigated the methylation status of the *PSEN1* gene in AD by employing a variety of approaches, including genome-wide methylation profiling and targeted methylation analysis, which have been implemented to investigate specific CpG sites or regions within the *PSEN1* gene. Monti and colleagues reported that the methylation status of *PSEN1* 5′ flanking was reduced in the cortex of AD patients Braak I–II and V–VI relative to age-matched controls. Remarkably, they also reported an inverse correlation between lower methylation and PSEN1 protein levels during the progression of the disease [59]. The hypomethylation status of *Psen1* has also been confirmed in TgCRND8 mice, a mouse model of AD [60]. The altered methylation status of the *PSEN1* gene in AD may have functional consequences for disease pathogenesis. Hypomethylation of the *PSEN1* promoter region may lead to an increase in gene expression and, consequently, increased PS1 protein levels. High PS1 levels can then increase Aβ production. 

#### 3.2.3. MAPT

**Figure 1:**
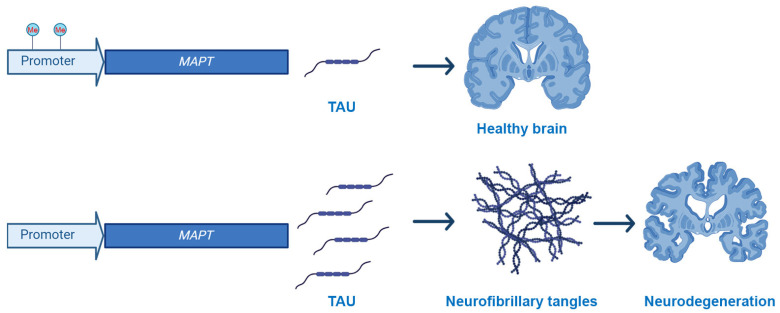
Schematic representation of the effects of methylation of the *MAPT* promoter on NFTs accumulation and neurodegeneration. Converging evidence indicates a link between hypomethylation of the *MAPT* promoter and increased tau production.

While there are no mutations in the *MAPT* gene that are associated with AD, several changes in its methylation profile have been published, even if often clear inconsistencies are reported in the literature (Figure 1). For example, in the frontal cortex and hippocampus, the percentage of CpG methylation of the *MAPT* gene was similar between AD patients and control cases [57]. Similarly, Mori and colleagues reported that the *MAPT* mRNA expression levels and methylation status were similar in the blood of AD and control cases [61]. However, hypomethylation of *MAPT* was observed in the temporal lobe of AD and was associated with higher tau expression and aggregation [60]. 

Another interesting aspect regulating tau function is the direct methylation of the tau protein, which in human brains can be mono- or di-methylated, while more aggregated tau is only mono-methylated [62], suggesting an inverse relation of tau with the extent of methylation [63]. To this end, Bichmann and colleagues reported that methylated tau is not associated with hyperphosphorylated tau [64]. Recently, it has been reported that tau methylation provides a signal for translocation to different subcellular compartments, specifically, methylated tau appears to be mainly localized in the cell soma [64]. This is interesting, considering that pathological tau is mislocalized from neurites to the cell bodies, where it accumulates. Moreover, physiological tau methylation in KXGS motifs reduces the phosphorylation potential on adjacent serine implicating the protective role of methylation [63]. Taken together, these results suggest that modulating methylation pathways linked to tau might be a promising avenue for research.

#### 3.2.4. APOE 

The human apolipoprotein E (*APOE*) gene, which is strongly associated with AD, has three alleles, E2, E3, and E4. The presence of a single copy of the E4 allele more than doubles the risk of developing AD, while the risk for homozygous E4 individuals is ~12-fold higher than for people homozygous for E3. In contrast, individuals with one or two E2 copies have a lower risk of developing the disease [1,2]. The exact mechanisms through which *APOE4* contributes to the development of AD are complex and not fully understood. However, strong evidence indicates that *APOE4* may influence the clearance of Aβ [65]. 

In recent years, a large body of evidence has indicated that in AD, the epigenetic mechanisms that control *APOE* expression might be altered. For example, a detailed analysis of the three *APOE* variants indicates that the base substitution in *APOE4* adds a new CpG site of methylation, while the base substitution in *APOE2* removes a CpG methylation site [Table 1 and [66]]. In addition, converging evidence indicates that changes in the *APOE* genotype alter the methylation state of other sites throughout the genome [67,68,69].

Overall, consistent results have shown that the *APOE* gene is significantly reduced in AD brains compared to age-matched controls [67,70]. It appears that this hypomethylation is mainly driven by changes in glia rather than neurons [71]. While it is unclear how these changes impact the role of the three different *APOE* isoforms on AD pathogenesis, Chang-En Yu and colleagues reported an inverse correlation between *APOE* methylation level and total *APOE* RNA in the frontal lobe of old, cognitively healthy people. However, this correlation was absent in age-matched AD cases [72]. Understanding the role of APOE4 in Alzheimer’s is crucial for advancing research and developing targeted interventions that may help mitigate the risk or progression of the disease in individuals with this genetic variant.

#### 3.2.5. IL1β

As discussed above, IL-1β and other cytokines released from activated microglia, contribute to Aβ production [8]. Several hypotheses have been proposed to explain the link between cytokines and Aβ. Nicolia et al. found that the promoter of the *IL1β* gene is hypomethylated early in disease progression, but the methylation state is unchanged when comparing people with advanced AD to age-matched cognitively normal people [73]. In contrast, they found that *IL6* methylation decreased with AD progression in the frontal cortex of AD compared to healthy controls [73]. These results may explain changes in IL1β and IL6 protein levels observed through the progression of the disease [74,75]. Further studies are needed to determine a causal relationship between methylation of the *IL1β* promoter and AD neuropathology. 

## 4. Histone Modification in AD

In chromatin, the DNA is wrapped around histone proteins to form a nucleosome. Histones control chromatin architecture, nucleosome positioning, and access of transcription factors and other DNA-binding proteins to the DNA. Heterochromatin and euchromatin refer to chromatin structures that are strongly or loosely packed around histones, respectively. Generally, regions of heterochromatin are less transcriptionally active than regions of euchromatin. However, posttranslational modifications (PTMs) of the amino-terminal tails of the histones regulate their interactions with DNA and thereby regulate gene expression. Physiologically, histone tails undergo methylation, acetylation, ubiquitylation, SUMOylation, glycosylation, and ADP-ribosylation [76].

### 4.1. Acetylation

A classic example of histone PTM is represented by acetylation. A family of enzymes known as histone acetyltransferases (HATs) can add acetyl groups to amino-terminal lysines of one or more core histones. This is a reversible reaction as histone deacetylases (HDACs) can remove the acetyl groups added by HATs. Overall, the presence of acetyl groups at the amino terminal of histones decreases the interaction of histone/DNA thereby “opening” the chromatin. In other words, by regulating the activity of HATs and HDACs, cells can control gene expression by making the DNA more or less accessible to DNA-binding proteins such as transcription factors [77]. 

In AD, dysregulation of histone acetylation results in changes in synaptic plasticity and memory processes [78,79] (Table 2). To this end, Santana and colleagues reported that hyperacetylation of the cerebellum and a slight hypoacetylation of the hippocampus of AD patients. These changes were associated with the activation of Rho GTPase-mediated mechanisms and cytoskeletal disorganization [80]. Similar results were obtained in mice; for example, Arancio and colleagues reported a 50% reduction of learning-induced acetylation of H4 in the hippocampus of APP/PS1 mice [79]. The positive effects of HDAC inhibitors on AD-like pathology have been confirmed by others in multiple animal models of AD (e.g., [81,82,83,84,85]). However, the use of HDAC inhibitors for the treatment of AD is hampered by the fact that most inhibitors are not selective and as such have notable side effects [86]. 

Among all HATs, CREB-binding protein (CBP), P300, or p300/CBP-associated factor (PCAF) are associated with long-term memory mechanisms. Notably, work from different laboratories has found that modulating the activity of these HATs has beneficial effects on AD-like pathology in mice. For example, Creighton and colleagues reported that PCAF activation improved memory loss in 3xTg-AD mice [87], a widely used animal model of AD. In the same animal model, we found that activity-dependent CREB activation was impaired in young 3xTg-AD mice. Restoring CREB function by increasing CBP expression rescued learning and memory deficits [88]. 

### 4.2. Histone Methylation/Demethylation

Histone methylation/demethylation is a reversible process involving the addition or removal of methyl groups to the N-terminal region of lysine or arginine residues. This process is mediated by histone methyltransferases (HMTs) and histone demethylases (HDMs), respectively. Changes in histone methylation are associated with changes in chromatin structure thereby modifying gene expression. Notably, arginine residues can undergo mono-methylation, whereas lysine residues can undergo mono-, di-, and tri-methylation. These modifications result in the activation or suppression of gene expression and have been linked to several physiological and pathological processes. For example, mice lacking the *Jmjd2B* gene in neurons, a histone demethylase specific for H3K9me3, have impaired working memory [89].

Extensive research has been conducted as to the changes in histone methylation and AD, which has been reviewed elsewhere, e.g., [90]. Multiple laboratories have reported changes in histone methylation status in AD (Table 2). For example, Anderson and Turko found a 25 and 35% reduction in H2B-methylation in residue K108 and R55, respectively, in the frontal cortex of AD brains [90]. While the number of AD brains in the study was limited, the results were convincing. Persico and colleagues confirmed lower H3K4me3 and higher H3K27me3 signals in AD patients compared to healthy individuals [91]. Multiple hypotheses have been proposed to link the reported changes in histone methylation to AD pathogenesis. Direct methylation of the *MAPT* gene has been associated with tau aggregation and neurodegeneration [92]. In addition, alteration in histone methylation can reduce autophagy function [93], thereby leading to AD neuropathology. Indeed, multiple reports indicate that autophagy regulates the turnover of Aβ and tau from the brain of AD [94]. Overall, more needs to be done to understand the causes and consequences of modification in histone methylation in AD, which may lead to the identification of novel therapeutic targets for future drug development.

### 4.3. Histone Phosphorylation 

Histone phosphorylation is another histone modification linked to the modulation of gene expression. It is mainly linked to chromatin remodeling, DNA repair, and cell death [95,96]. A well-characterized phosphorylation event is associated with histone H3 phosphorylation. This post-translational modification has been linked to learning and memory pathways [97]. For example, phosphorylation of Ser28 of histone H3 regulates the expression of *cFOS*, an immediate early gene associated with memory formation and consolidation [95,98]. In AD, Chaput and colleagues showed that phosphorylation levels of Ser 47 of histone H4 (H4S47p) positively correlate with AD progression. Consistent with these observations, phosphorylation of histone H3 is also increased in the frontal cortex of AD [32] (Table 2). 

Following double-strand breaks, cells, including neurons, express a variant of histone H2A known as H2AX, which can be phosphorylated at Ser139. The levels of H2AX at Ser 139 are increased in cortical and hippocampal astrocytes of AD patients compared to control individuals [96]. While future studies are needed to fully elucidate the link between phosphorylated H2AX and AD, these data underscore a novel link between astrocytes and AD.

Although different hypotheses have been formulated, more needs to be done to understand the mechanisms underlying neuronal death in AD [99,100,101]. A prevalent hypothesis states that in AD, neurons attempt to reenter the cell cycle, and this process leads to their death, e.g., [101]. This idea is supported by the increase in the levels of histone H3 phosphorylated at Ser10 in the cytoplasm of hippocampal AD neurons [102]. Indeed, the presence of cytosolic histones is an indication of cell cycle reactivation.

As we discussed above, often there is no concordance between various studies when it comes to selective epigenetic changes. This appears not to be the case for phosphorylation of various histones. Future studies will have to dissect the molecular pathways linking histone phosphorylation and AD.

### 4.4. Histone Ubiquitylation

Lysine ubiquitylation is a posttranslational modification of histones, where a ubiquitin molecule is attached to specific lysine residues. It can have diverse effects on chromatin structure and function, depending on the specific lysine residue modified and the number of ubiquitin molecules added. For example, lysine ubiquitylation can either promote or inhibit gene expression, depending on which lysine residue is ubiquitylated. In addition, this posttranslational modification can alter chromatin structure by affecting the interactions between histones and DNA, as well as the interactions between neighboring nucleosomes. This can influence higher-order chromatin organization and dynamics. Another major consequence of lysine ubiquitylation is the regulation of epigenetic inheritance. To this end, it can serve as a marker for epigenetic inheritance, influencing the transmission of chromatin states through cell divisions and potentially across generations [103]. Dysregulation of histone ubiquitylation has been implicated in various human diseases, including neurodegenerative disorders. To this end, Anderson and Turko showed that in the frontal cortex of AD cases, there was a striking 91% increase in ubiquitination of K120 on H2B [90] (Table 2). In an independent study, Guo and colleagues showed that in the cortex of AD cases, there was a ~50% increase in H2A ubiquitination of K119 and a ~2-fold increase in the combined levels of mono- and penta-ubiquitinated H2A [104]. While the results are striking, it should be noted that in both cases, the number of AD brains analyzed was undoubtedly small (6 and 7, respectively). Thus, further studies are required to establish a conclusive link between histone ubiquitination and AD.

**Table 2:** Summary of the major histone modification identified in human AD and mouse models.

Type	Site	Regulation	Proposed Effects	Site of the Analysis	Tissue	Ref.
Acetylation	Lysine 9 of H3	increase	Rho GTPase-mediated mechanism activation; cytoskeletal disorganization	Cerebellum	AD patients	[80]
		decrease		Hippocampus		
	Total acetylation levels of H4	decrease	Reduction of learning	Hippocampus	APP/PS1 mice	[79]
	Total acetylation levels of H3/H4	decrease	Deficit of cognitive function related to altered hippocampal gamma oscillations	Hippocampus	PSAPP mice	[85]
Methylation	Lysine 108 of H2B and arginine 55 of H4	decrease	Altered nucleosome stability by hydrogen bonding networks	Frontal cortex	AD patients	[90]
	Lysine 4 of H3	decrease	heterochromatinization expansion of encoding regions of the genomes associated with neurodegeneration	Entorhinal cortices	AD patients	[91]
	Lysine 27 of H3	increase				
Phosphorylation	Total level phosphorylation of H3	increase	Positive correlation with AD progression	Frontal cortex	AD neurons	[32]
	Serine 139 of H2AX variant			Cortical and hippocampal astrocytes		[96]
	Serine 10 of H3			Cytoplasm of hippocampal		[102]
Ubiquitylation	Lysine 120 of H2B	increase	Altered nucleosome stability by hydrogen bonding networks	Frontal cortex	AD patients	[90]
	Lysine 229 of H2A		Cellular senescence and proteasome-mediated histone H2A proteolysis	Cortex		[104]

## 5. Therapeutic Opportunities

Mammals have four classes of HDACs. Class I includes HDAC1, HDAC2, HDAC3, and HDAC8. They are primarily localized in the nucleus and are involved in regulating gene expression through histone deacetylation. Class II is further subdivided into two subclasses: IIa and IIb. The latter includes HDAC4, HDAC5, HDAC7, and HDAC9. They shuttle between the nucleus and cytoplasm and are involved in processes such as muscle differentiation and development. The former include HDAC6 and HDAC10, which are localized in the cytoplasm. Class III, also known as sirtuins, includes SIRT1–7. Notably, the HDACs in this class require NAD+ as a cofactor for their deacetylase activity. Class IV includes only HDAC11. Its structure resembles both Class I and II HDACs, and its functions are still being elucidated (see [105] for structural details about the four classes).

Several laboratories have reported the positive effects of HDAC inhibitors on multiple animal models of AD. For example, Arancio and colleagues, in an early report, highlighted how acute administration of trichostatin A (TSA) restored the levels of H4 acetylation in APP/PS1 mice. These changes were associated with improved hippocampal long-term potentiation and contextual memory [79]. These findings were confirmed in a different mouse model and with a different dosing paradigm. Specifically, Garcia-Osta and colleagues reported that sodium 4-phenylbutyrate (4-PBA) administered daily for five weeks to 16-month-old Tg2576 mice restored the levels of H4 acetylation while improving learning and memory [106]. Since these two publications, others have found similar results using yet other mouse models of AD and, more relevant to the topic at hand, more selective HDAC inhibitors [107,108,109]. While the literature consistently shows the beneficial effects of HDAC inhibitors on AD-like pathology in mouse models of AD, it should be noted that a few reports have also reported the lack of effects of a pan-specific HDAC inhibitor in transgenic AD mice. For example, Hanson and colleagues reported that SAHA, when peripherally administered, showed reduced brain permeability and a lack of effects on cognition [110]. 

More recently, a great deal of attention has been given to HDAC inhibitors that are selective for specific classes of HDAC (reviewed in [111]). For example, in an early work, Green and colleagues dosed 3xTg-AD mice with nicotinamide, a sirtuin inhibitor, for four months. Nicotinamide was added to the mice’s drinking water before the onset of AD-like pathology. The authors reported that this experimental paradigm prevented memory deficits while decreasing tau pathology ([112]). The beneficial effects of nicotinamide have been replicated by a different group in the same animal model [113]. Unfortunately, a small double-blind randomized clinical trial (24 patients on nicotinamide and 22 placebo) failed to show significant changes in CSF tau and Aβ (ClinicalTrials.gov Identifier: NCT03061474 accessed on 20 April 2024).

Over the last several years, sirtuins, a family of enzymes with deacetylase activity, have taken center stage for their effects on aging and neurological disorders [114]. Mammals have 7 sirtuin genes: *SIRT 1* and *SIRT 2* are located in the nucleus and cytoplasm, *SIRT 3*, *SIRT 4*, and *SIRT 5* in the mitochondria, *SIRT 6* and *SIRT 7* in the nucleus ([115]). Sirtuins possess the ability to remove acetyl or acyl groups from both histone and non-histone proteins, including some transcription factors. Additionally, Sirt4 and Sirt6 exhibit ADP-ribosyltransferase activity, allowing them to transfer ADP-ribose from NAD+ to protein substrates. This broad spectrum of protein modification enables sirtuins to influence diverse cellular processes, including but not limited to energy metabolism, aging, and neurodegeneration [114]. Overwhelming evidence highlights a link between sirtuins and AD. In postmortem human AD brains, Julien and colleagues reported a significant decrease in the mRNA and protein levels of SIRT1 and an inverse correlation between reduced SIRT1 levels and high cortical Aβ and tau levels [116]. Converging preclinical data indicate a crosstalk between Aβ/tau and sirtuins; increasing Aβ or tau levels reduced sirtuins while increasing sirtuins expression decreases Aβ and tau (reviewed in [115]). 

Numerous preclinical studies have shown how molecules that increase sirtuin activity may have beneficial effects for AD. Among these, resveratrol has been tested by multiple laboratories yielding consistent positive results (reviewed in [117]). However, the results in clinical trials have not been so exciting. Over the 52-week trials in a double-blind, randomized, placebo-controlled phase 2 clinical trial in patients with mild to moderate AD, resveratrol appeared to increase brain volume loss compared to placebo [118]. It has been proposed that the increase in brain volume might be due to the anti-inflammatory effects of resveratrol and the reduction in CNS edema [119]. While some results appear promising, larger studies are needed to better define the role of resveratrol in AD. 

Overall, it appears evident that the lack of isoform specificity and the systemic distribution of some HDAC inhibitors contribute to safety concerns that have been raised over the chronic use of this class of inhibitors to treat AD. In addition, many HDAC inhibitors have limited blood–brain barrier permeability, which hampers their ability to reach a therapeutic concentration in the brain and may necessitate high doses that increase the risk of systemic side effects.

## 6. Concluding Remarks

This comprehensive review underscores the critical role of DNA methylation and histone modification in the intricate landscape of AD pathogenesis. The devastating impact that this disorder has on cognitive function and the pressing need for effective therapeutic interventions highlight the urgency of identifying the molecular mechanisms underlying its pathogenesis. As highlighted in this review, alterations in DNA methylation and histone modification have revealed their profound influence on key pathological events associated with AD. The dynamic interplay between genetic and environmental factors shaping the epigenetic landscape adds complexity to our understanding of the disease. Importantly, these epigenetic alterations extend beyond mere gene expression modulation, reaching into the dysregulation of fundamental cellular processes such as synaptic plasticity, neuroinflammation, and oxidative stress. The knowledge gleaned from this review not only deepens our comprehension of AD but also opens new avenues for targeted therapeutic strategies. As new data continue to bridge the gap between epigenetics and AD, it is increasingly clear that targeting epigenetic mechanisms may lead to the identification of innovative approaches that may ultimately lead to more effective treatments.

## Figures and Tables

**Table 1:** The three different *APOE* isoforms. Compared to APOE3, the single nucleotide changes in *APOE2* and *APOE4* lead to a decrease and increase of a methylation site, respectively.

Isoforms	Codon 112	Codon 158	CpG
*APOE2*	TGC	TGC	−1
*APOE3*	TGC	CGC	//
*APOE4*	CGC	CGC	+1
